# Socioeconomic Correlates of Sedentary Behavior in Adolescents: Systematic Review and Meta-Analysis

**DOI:** 10.1007/s40279-016-0555-4

**Published:** 2016-06-03

**Authors:** Gregore I. Mielke, Wendy J. Brown, Bruno P. Nunes, Inacio C. M. Silva, Pedro C. Hallal

**Affiliations:** 1 Postgraduate Program in Epidemiology, Federal University of Pelotas, Rua Marechal Deodoro 1160, 3rd floor, Pelotas, 96020-220 Brazil; 2 School of Human Movement and Nutrition Sciences, University of Queensland, Brisbane, QLD Australia; 3 Department of Nursing, Federal University of Pelotas, Pelotas, Brazil

## Abstract

**Background:**

The body of evidence on associations between socioeconomic status (SES) and sedentary behaviors in adolescents is growing.

**Objectives:**

The overall aims of our study were to conduct a systematic review and meta-analysis of this evidence and to assess whether (1) the associations between SES and sedentary behavior are consistent in adolescents from low-middle-income and from high-income countries, (2) the associations vary by domain of sedentary behavior, and (3) the associations vary by SES measure.

**Methods:**

We performed a systematic literature search to identify population-based studies that investigated the association between SES and sedentary behavior in adolescents (aged 10–19 years). Only studies that presented risk estimates were included. We conducted meta-analyses using random effects and univariate meta-regression and calculated pooled effect sizes (ES).

**Results:**

Data from 39 studies were included; this provided 106 independent estimates for meta-analyses. Overall, there was an inverse association between SES and sedentary behavior (ES 0.89; 95 % confidence interval [CI] 0.81–0.98). However, the direction of the association varied: in high-income countries, SES was inversely associated with sedentary behavior (ES 0.67; 95 % CI 0.62–0.73), whereas in low-middle-income countries, there was a positive association between SES and sedentary behavior (ES 1.18; 95 % CI 1.04–1.34). In high-income countries, the associations were strongest for screen time (ES 0.68; 95 % CI 0.62–0.74) and television (TV) time (ES 0.58; 95 % CI 0.49–0.69), whereas in low-middle-income countries, the associations were strongest for ‘other’ screen time (i.e., computer, video, study time, but not including TV time) (ES 1.38; 95 % CI 1.07–1.79). All indicators of SES were negatively associated with sedentary behavior in high-income countries, but only resources (income and assets indexes) showed a significant positive association in low-middle-income countries.

**Conclusion:**

The associations between SES and sedentary behavior are different in high- and low-middle-income countries, and vary by domain of sedentary behavior. These findings suggest that different approaches may be required when developing intervention strategies for reducing sedentary behavior in adolescents in different parts of the world.

**Electronic supplementary material:**

The online version of this article (doi:10.1007/s40279-016-0555-4) contains supplementary material, which is available to authorized users.

## Key Points

**Table:** 

Associations between socioeconomic status (SES) and sedentary behavior differ between adolescents from high- and low-middle-income countries and vary by domain of sedentary behavior.
In high-income countries, there was a strong and consistent inverse association between SES and total screen time and television (TV) time. In contrast, in low-middle-income countries, SES was not associated with total screen or TV time, but there was a positive association between SES and “other screen time” (i.e., video, computer games, or study time, but not including TV time).
This review suggests the use of different approaches in low-middle- and high-income countries for reducing sedentary behavior in adolescents.

## Introduction

During the last decade, the concept of being ‘sedentary’ has changed. Whereas it was once understood as not meeting the guidelines for moderate to vigorous physical activity [1], the term ‘sedentary behavior’ is now used to describe waking behaviors that involve sitting or lying down [2]. Although the independent effects of sedentary behavior and physical activity in terms of health consequences are debated, there is consensus that the correlates of these behaviors may be different, in both adolescents and adults [3, 4].

Three recent reviews have shown that socioeconomic status (SES) is an important correlate of sedentary behavior, and that children and adolescents from lower socioeconomic backgrounds have higher levels of sedentary behavior, in both screen-based and non-screen-based activities [4–6]. In contrast, a systematic review of the correlates of sedentary behavior among school-aged children in Sub-Saharan Africa found that higher SES was associated with more sedentary behavior [7].

As correlates of different domains of sedentary behavior (such as television [TV] time, screen time, studying, etc.) are likely to differ, some studies focused on only one sedentary behavior domain [6]. However, others grouped time spent in different domains [4, 7] making it difficult to assess domain-specific correlates. Moreover, many studies focused on only one indicator of SES: either parental income, occupation, or education [8–12]. However, it is possible that, although SES measures are strongly correlated, they might influence health behavior differently [13, 14]. For example, while resources (e.g., income or assets index) might be strongly related to ownership of electronic devices at home (thereby allowing access to “screen” devices), parental education might be associated with parental rules limiting access to these devices [15, 16].

One limitation of much of the research to date is that most studies have focused on both children and adolescents [5, 6]. However, the correlates of sedentary behavior may differ in children and adolescents (defined by the World Health Organization as aged 10–19 years) [17], because of increasing autonomy for decision making as young people move through their teenage years. To inform the development of effective interventions for reducing sedentary behaviors in adolescents, it is important to understand the socioeconomic determinants of the different domains of sedentary behavior at this specific life stage.

The aims of this review were, via meta-analysis, to examine the SES correlates of sedentary behavior in adolescents, and to examine whether (1) the associations between SES and sedentary behaviors are consistent in adolescents from low-middle-income and from high-income countries, (2) the associations vary by domain of sedentary behavior, and (3) the associations vary by SES measure.

## Methods

### Search Strategy

In 2015, we conducted a systematic search in the Academic Search Premier, CINAHL, Cochrane, PubMed, Scopus, SocIndex, SPORTDiscus, and Web of Science databases to identify relevant studies on associations between SES and sedentary behavior in adolescents. Groups of thesaurus terms and free terms were searched using a Boolean strategy: terms for adolescents (“adolescence” OR “adolescent” OR “adolescents” OR “teen” OR “teenager” OR “teenagers” OR “teens” OR “youth” OR “youths”) were used in AND combination with terms for sedentary behavior (“Sedentary behavior” OR “Sedentary behaviour” OR “Sedentary time” OR “Sitting time” OR “Television” OR “Screen-based” OR “TV viewing” OR “Computer use”) AND terms for socioeconomic status (“Schooling attainment” OR “Family income” OR “income” OR “Socioeconomic position “OR “Socioeconomic level” OR “Economic level” OR “Assets index” OR “Poverty” OR “Deprivation” OR “Schooling” OR “education” OR “disparity” OR “ethnic” OR “inequality” OR “inequity” OR “race”). All studies published up to 19 March 2015 were considered.

### Eligibility and Exclusion Criteria

We considered only full-text, peer-reviewed population-based studies focusing on adolescents (mean age 10–19 years) [17], with a measure of SES as the exposure, and a measure of sedentary behavior as the outcome, and reporting an association between SES and sedentary behavior variables. Measures of parental education, income, assets index/deprivation, and occupation were considered as indicators of SES. The search was restricted to studies published in English, Portuguese, or Spanish. Review papers, theses, and dissertations were not included.

We applied the following exclusion criteria:

(i) sedentary behavior was inappropriately defined, i.e., defined as not meeting physical activity guidelines;
(ii) the focus was on a specific clinical population (e.g., overweight or obese, people with Down syndrome or other disability; people with a specific illness);
(iii) there was no heterogeneity in socioeconomic level, i.e., only those in a specific socioeconomic level were included;
(iv) the study was an intervention that aimed to reduce sedentary behavior (with the exception of studies reporting on baseline data from intervention studies);
(v) sedentary time was an exposure instead of an outcome measure;
(vi) the study included children, adolescents, and adults, but did not present separate analyses for adolescents. In studies that included children and adolescents, but did not present separate analyses for adolescents, studies were excluded if the average age was <10 or >19 years (or where the majority of participants were not aged between 10 and 19 years);
(vii) the study did not provide data on the association between SES and sedentary behavior, from analyses of primary or secondary data (or did not provide data to enable calculation of these estimates, for example, from 2 × 2 tables).

### Data Extraction

The first author (GIM) conducted the search; two independent reviewers (GIM and BPN) evaluated all abstracts. If the two reviewers were unsure, they sought consensus from all authors. Three independent reviewers (GIM, BPN, and ICMS) extracted information from all the included papers, and any disagreement was resolved by consensus in consultation with the other authors (WJB and PCH). Extracted information included authors, year of publication, country in which the study was conducted, survey year, study design, sample size, age range, type(s) of SES measures, number of SES categories, and sedentary behavior domains and definitions.

Where reported, odds ratio (OR) and respective standard errors or 95 % confidence intervals (CIs) were extracted. If these data were not reported or could not be calculated, we contacted the first author of the study via email. If the authors could not be contacted, or could not supply the data, we excluded the study. We also excluded studies that only presented sedentary behavior as a continuous variable, and did not report a categorical variable for “high” sedentary behavior.

To prevent duplication, if multiple publications were available from the same data source/study population, we used the most recent or most complete data. In cases where publications had complementary information (i.e., one provided data about one sedentary domain and/or SES measure and another provided data about other associations) we included both studies. If studies reported findings for boys and girls separately, we included two independent estimates in the meta-analysis. If studies measured sedentary behavior separately on weekdays and weekends, we only used estimates from weekdays.

The manuscript was modelled on the Preferred Reporting Items for Systematic Reviews and Meta-Analyses (PRISMA) Statement [18].

### Data Management and Statistical Analysis

Initially, we performed a general meta-analysis, with pooling of all estimates, using the original sedentary behavior domains (study, TV, video games, personal computer, screen time, or combinations of these) and SES measures (paternal, maternal, or parental education; assets index/deprivation; income; paternal, maternal, or parental occupation). We then performed a series of univariate meta-regressions to investigate the main sources of heterogeneity in the overall meta-analysis (see Electronic Supplementary Material [ESM] Table S1). For these analyses, SES measures were categorised as (1) education (paternal, maternal or parental education); (2) resources (including income, assets index, and deprivation); or (3) occupation (paternal, maternal, or parental education). Sedentary behaviors were categorised as (1) screen-based (for studies that considered TV time and/or video game time and/or computer time together; (2) TV-viewing time (for studies that measured only TV time); or (3) “other” (for studies that measured computer and/or video game time and/or time spent studying, but not TV time). Data were stratified by the World Bank’s country classification (low- or middle-income country; or high-income country). We used random-effects models to calculate pooled effects sizes (ES) and assessed heterogeneity using the I-squared test.

In all the analyses, we used the lowest SES category as the reference group. Thus, effect measures higher than 1.00 indicate more sedentary behavior, and effect measures lower than 1.00 indicate less sedentary behavior in higher SES groups than in the reference (low) SES group. Comparisons of the highest and the lowest socioeconomic groups reported in each paper were included in the meta-analysis.

We conducted several sensitivity analyses to assess the robustness of the data and to explore potential sources of heterogeneity. These analyses included (1) data from studies that reported only one SES variable; (2) a comparison of studies that used two and those that used more than two SES categories; (3) exclusion of studies in which the reference category was changed to allow inclusion of the data; (4) exclusion of studies that included participants who were aged <10 or >19 years; (5) exploration of bias due to different definitions of “high” sedentary behavior, and (6) analysis of data from studies that did not stratify by sex. We used funnel plots and Egger tests to investigate publication bias.

## Results

**Fig. 1:**
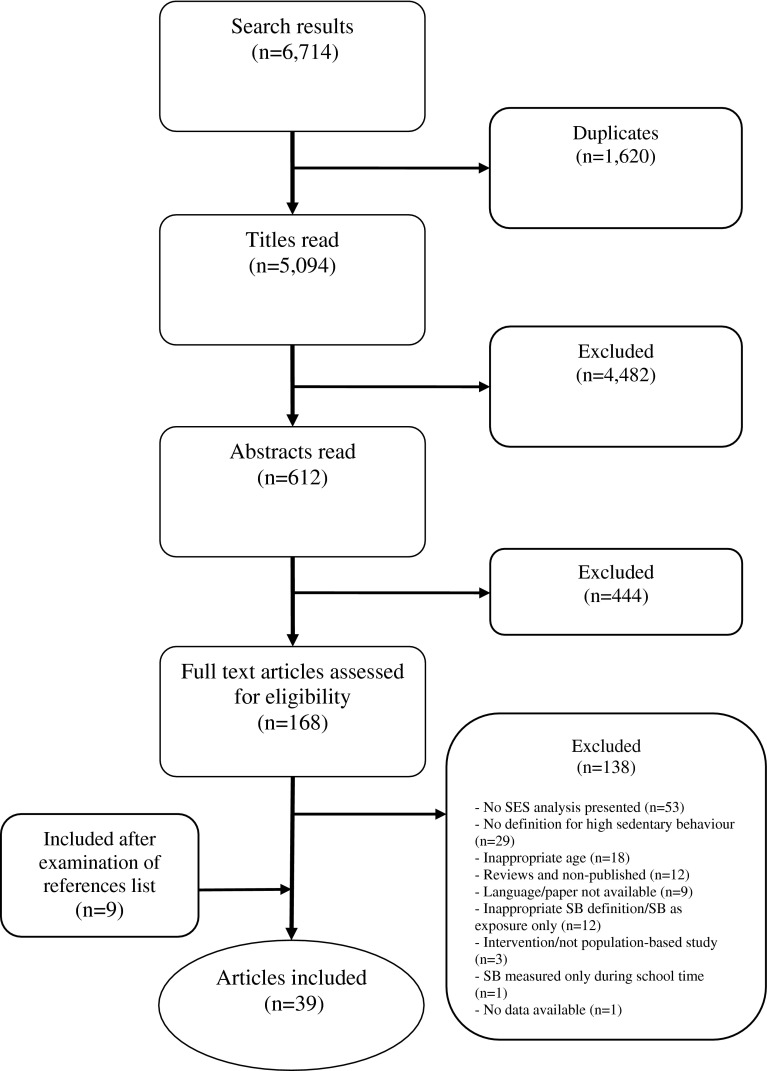
Flowchart reporting the process for selection of papers for inclusion in the meta-analysis. *SB* sedentary behavior, *SES* socioeconomic status

Figure 1 provides an overview of the search process. We identified 6174 references, 612 of which were identified as potentially relevant after exclusion of duplicates and those that did not meet the inclusion criteria. Of these, 444 were excluded after abstract review, mostly because they did not report an association between SES and sedentary behavior. After full review of the remaining 168 papers, 39 were considered for inclusion in the meta-analysis. These papers included 106 separate estimates of SES–sedentary time associations.

Table 1 shows the characteristics of the included studies, which were from 15 different countries: Brazil (=12), the USA (=8), Australia (=4), China (=3), England (=3), and Norway (=2) contributed more than one study, and the remaining nine countries contributed one each. All were conducted between 1994 and 2011 and published between 2000 and 2015; most were of cross-sectional design. Sample size ranged from small studies of <500 adolescents in Australia and France to large studies of >50,000 participants in Brazil and the USA. The age range was from 6 to 19 years, with average age between 10 and 19 in all studies. The 39 papers included nine measures of SES, including paternal, maternal, or parental education (25 studies), resources (23 studies), and parental occupation (five studies). The majority (*n* = 23) considered only one measure of SES, but five studies included three or more measures of SES. The majority of studies (*n* = 34) also used a single measure of sedentary behavior; this was most commonly TV time (*n* = 17) or a composite measure of time watching TV and playing video games or using a computer (*n* = 10). Three studies measured video game and computer time separately, and 15 measured total screen time. Most studies (*n* = 32) presented analyses for boys and girls combined; only seven presented separate analyses for boys and girls (Table 1).

**Table 1:** Descriptive characteristics of the included study

Study	Country	Survey year	Study design	Sample size	Age range (years)	SES measures	Definition of sedentary behavior
Al Sabbah et al. [25]	Palestine	2004	Cross-sectional	8885	12–18	Paternal education, maternal education	TV (≥4 h/day), PC use, homework (≥4 h/day)
Atkin et al. [8]	Denmark	1997–1998	Cross-sectional	1746	8–16	Maternal education	TV + PC (≥2 h/day)
	Estonia	1998–1999		652	8–17		
	England	2006–2009		2154	9–12		
Barbosa Filho et al. [20]	Brazil	2011	Cross-sectional	1628	11–17.9	Parental education, assets index	TV (≥3 h/day)
Camelo et al. [29]	Brazil	2009	Cross-sectional	59,809	13–16	Assets index	TV (>2 h/day)
Carlson et al. [30]	USA	2004–2006	Cross-sectional	7415	9–15	Parental education, income	Screen time (≥2 h/day)
Cui et al. [31]	China	2006	Cross-sectional	1128	6–18	Income	Screen time (≥2 h/day)
da Silva et al. [32]	Brazil	2001–2002	Cross-sectional	5028	15–19	Income	Screen time (≥4 h/day)
Dias et al. [33]	Brazil	2009–2011	Cross-sectional	1716	10–17	Paternal education, maternal education, assets index	Screen time (≥4 h/day)
Dumith et al. [10]	Brazil	2004	Cross-sectional	4431	11	Assets index	Screen time (≥4 h/day)
Fernandes et al. [34]	Brazil	2007	Cross-sectional	1752	11–17	Assets index	TV (very often)
Foltz et al. [11]	USA	1999–2002	Cross-sectional	4414	12–19	Income	TV + PC (≥2 h/day)
Gordon-Larsen, et al. [35]	USA	1995	Cross-sectional	17,776	11–21	Maternal education, income	Screen time (≥25 h/week)
Hallal et al. [9]	Brazil	2009	Cross-sectional	59,809	13–16	Maternal education	TV (≥2 h/day)
Hardy et al. [27]	Australia	2002–2003	Cross-sectional	343	12–13	Maternal education	TV (>2 h/day)
Hardy et al. [36]	Australia	2004	Cross-sectional	2750	11–15	Assets index, deprivation	Screen time (≥2 h/day)
Jiang et al. [37]	China	2011	Cross-sectional	3461	12–14	Paternal education, maternal education	Screen time (≥2 h/day)
Kantomaa et al. [38]	Finland	2001–2002	Cross-sectional	5085	16	Paternal education, maternal education, income	TV (≥3.5 h/day)
Kiatrungrit et al. [39]	Thailand	2011	Cross-sectional	768	11–19	Paternal education, maternal education	TV (≥3 h/day), VG (≥3 h/day), PC (≥3 h/day), mobile phone (≥3 h/day), electronic devices (≥12 h/day)
Kim et al. [40]	USA	1994–1995	Cross-sectional	13,668	15.8^a^	Parental education	Screen time (≥2 h/day)
Kipping et al. [28]	England	2006–2008	Longitudinal	6406	15–16	Maternal education, maternal income, parental occupation	TV (≥3 h/day)
Kristiansen et al. [41]	Norway	2004–2006	Cross-sectional	2281	6–15	Parental education	TV + PC (≥2 h/day)
Lioret et al. [42]	France	1998–1999	Cross-sectional	333	11–14	Parental occupation	TV + VG (≥1.2 h/day)
Mutunga et al. [43]	Northern Ireland	2000	Cross-sectional	2016	12–15	Parental occupation	TV + PC (≥4 h/day)
Norman et al. [44]	USA	NA	Cross-sectional	878	11–15	Parental education	Screen time (≥4 h/day)
Ogunleye et al. [45]	England	2007–2009	Cross-sectional	6240	10–16	Assets index/deprivation	Screen time (≥2 h/day)
Øverby et al. [46]	Norway	2001–2008	Surveillance	2001: 1488	10–12	Parental education	TV + PC (≥2 h/day)
				2008: 1339			
Patriarca et al. [47]	Italy	2007	Cross-sectional	987	11–16	Parental occupation	TV (≥2 h/day)
Rey-Lopez et al. [23]	Spain	2002	Cross-sectional	1776	13–18	Paternal education, maternal education, paternal occupation, maternal occupation	TV (≥3 h/day), VG + PC (≥1 h/day), studying (≥3 h/day)
Rivera et al. [48]	Brazil	2001	Cross-sectional	1253	12.4^a^	Assets index	TV (≥3 h/day)
Salmon et al. [12]	Australia	2001	Cross-sectional	1756	10–12	Maternal education	TV (>2 h/day)
Shi et al. [24]	China	2002	Cross-sectional	824	12–14	Paternal education, assets index	TV + VG (>1 h/day), study
Silva et al. [21]	Brazil	2011	Cross-sectional	2105	13–18	Paternal education, maternal education, assets index	TV (≥2 h/day)
Silva et al. [49]	Brazil	2001–2011	Surveillance	2001: 5028	15–19	Income	TV (>2 h/day), VG + PC (>2 h/day)
				2011: 6529			
Singh et al. [50]	USA	2003–2004	Cross-sectional	68,288	6–17	Parental education, income	TV + VG (≥3 h/day)
Sisson et al. [51]	USA	2003–2004	Cross-sectional	33,117	6–17	Parental education, income	TV + VG (≥2 h/day)
Sisson et al. [52]	USA	2007–2008	Cross-sectional	48,505	6–17	Income	TV + VG (≥2 h/day)
Smith et al. [22]	Australia	2007	Cross-sectional	1685	13.6^a^	Maternal education, income	Screen time (≥2 h/day)
Tenorio et al. [53]	Brazil	2006	Cross-sectional	4210	14–19	Maternal education	TV (≥3 h/day)
Wells et al. [54]	Brazil	2004	Longitudinal	4289	11	Maternal education, assets index	TV (>2 h/day)

*NA* not available, *PC* computer, *SES* socioeconomic status, *TV* television, *VG* video game

^a^Mean age

**Fig. 2:**
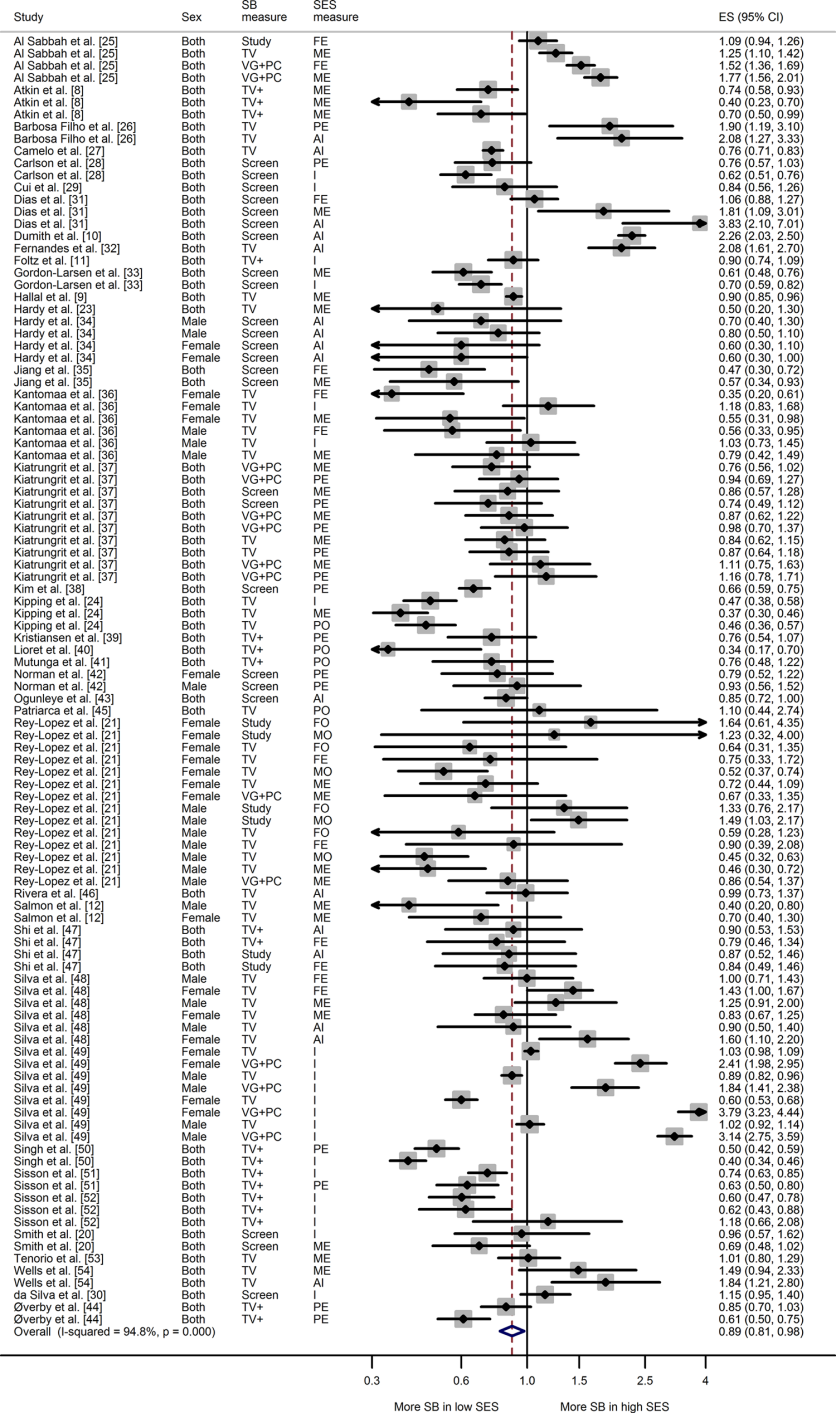
General meta-analysis of the association between socioeconomic status and sedentary behavior. *AI* assets index, *CI* confidence interval, *ES* effect size, *FE* paternal education, *FO* paternal occupation, *I* income, *ME* maternal education, *MO* maternal occupation, *PC* computer, *PE* parental education, *PO* parental occupation, *SB* sedentary behavior, *SES* socioeconomic status, *TV* television, *VG* video game. *TV* *+* indicates estimates based on studies that measure sedentary behavior as: **a** TV + VG or **b** TV + PC

The results of the overall meta-analysis (Fig. 2) showed the odds of high sedentary behavior were 11 % lower in the highest SES groups than in the lowest SES groups (ES 0.89; 95 % CI 0.81–0.98). As expected, there was substantial heterogeneity among studies (*I* ^2^ = 94.8 %); the sources of this are described below.

Table 2 presents the results of the meta-regression analyses of heterogeneity sources. The top panel of Table 2 shows the main source of heterogeneity (*R* ^2^ = 37.3) was country income status; there was a negative association between SES and sedentary behavior in adolescents from high-income countries (ES 0.67; 95 % CI 0.62–0.73) and a positive association in studies from low-middle-income countries (ES 1.18; 95 % CI 1.04–1.30). There was also some heterogeneity (*R* ^2^ = 20.2) in terms of the domain of sedentary behavior, with negative associations for screen and TV time and a positive association for “other” screen time. Heterogeneity due to the SES measure was low (*R* ^2^ = 6.9).

**Table 2:** General meta-analysis showing heterogeneity sources, and meta-regression of the associations between socioeconomic status and high sedentary behavior variables in low-middle-income and high-income countries (106 estimates from 39 studies)

Variables	*n* ^a^	ES pooled (95 % CI)	*I* ^2^	Meta-regression OR (95 % CI)	% Heterogeneity explained (*R* ^2^)
General					
Sedentary behavior definition					
Screen time^b^	42	0.77 (0.66–0.90)	93.6	Index	20.2
TV	43	0.86 (0.77–0.95)	90.0	1.11 (0.91–1.35)	
Other^c^	21	1.32 (1.06–1.66)	94.0	1.74 (1.37–2.21)	
SES					
Education	38	0.82 (0.74–0.91)	89.3	Index	6.9
Resource	56	1.06 (0.89–1.26)	97.3	1.30 (1.06–1.58)	
Occupation	12	0.73 (0.53–1.00)	78.1	0.89 (0.64–1.25)	
Country income^d^					
Low-middle income	49	1.18 (1.04–1.34)	96.0	Index	37.3
High-income	57	0.67 (0.62–0.73)	74.9	0.57 (0.49–0.67)	
Low-middle-income countries					
Sedentary behavior definition					
Screen time^b^	12	1.06 (0.76–1.47)	93.5	Index	4.3
TV	22	1.08 (0.97–1.20)	89.8	1.06 (0.75–1.48)	
Other^c^	15	1.38 (1.07–1.79)	95.6	1.31 (0.91–1.89)	
SES					
Education	28	1.04 (0.92–1.17)	86.2	Index	10.8
Resource	21	1.42 (1.13–1.79)	98.0	1.38 (1.07–1.78)	
Occupation	0	–	–	–	
High-income countries					
Sedentary behavior definition					
Screen time^b^	30	0.68 (0.62–0.74)	73.8	Index	28.9
TV	21	0.58 (0.49–0.69)	67.0	0.85 (0.71–1.02)	
Other^c^	6	1.15 (0.87–1.52)	22.7	1.69 (1.22–2.34)	
SES					
Education	28	0.63 (0.57–0.70)	57.8	Index	–3.3
Resource	17	0.72 (0.61–0.84)	85.0	1.13 (0.93–1.38)	
Occupation	12	0.73 (0.53–1.00)	78.1	1.11 (0.86–1.44)	
Total	106	0.89 (0.81–0.98)	94.8	–	–

*CI* confidence interval, *ES* effect size, *OR* odds ratio, *SES* socioeconomic status

^a^Represents the number of estimates available

^b^Estimates based on studies that measured sedentary behavior as time spent in TV + computer + video games + other screen-based activities

^c^Estimates based on studies that measured sedentary behavior as time spent in computer, video game, study time, but not including TV time

^d^According to World Bank classification

When the data were stratified by country income (middle and lower panels of Table 2), the analyses showed that sedentary behavior domains explained 29 % of the heterogeneity in high-income countries, but only 4.3 % in low-middle-income countries. The association between SES and high screen and TV time was negative in high-income countries, indicating lower odds of high sedentary behavior in the highest than in the lowest SES groups. In low-middle-income countries, only the association between SES and “other” screen time was significant; there was a positive relationship, indicating greater likelihood of high “other” screen time in high SES than in low SES groups.

The way SES was measured was more important in low-middle-income countries (*R* ^2^ = 10.8) than in high-income countries (*R* ^2^ = −3.3 %). Among low-middle-income countries, resource measures were more strongly associated with sedentary behaviors than measures related to parental education (OR_meta-regression_ 1.38; 95 % CI 1.07–1.78). This pattern was not observed in high-income countries (OR_meta-regression_ 1.13; 95 % CI 0.93–1.38).

**Fig. 3:**
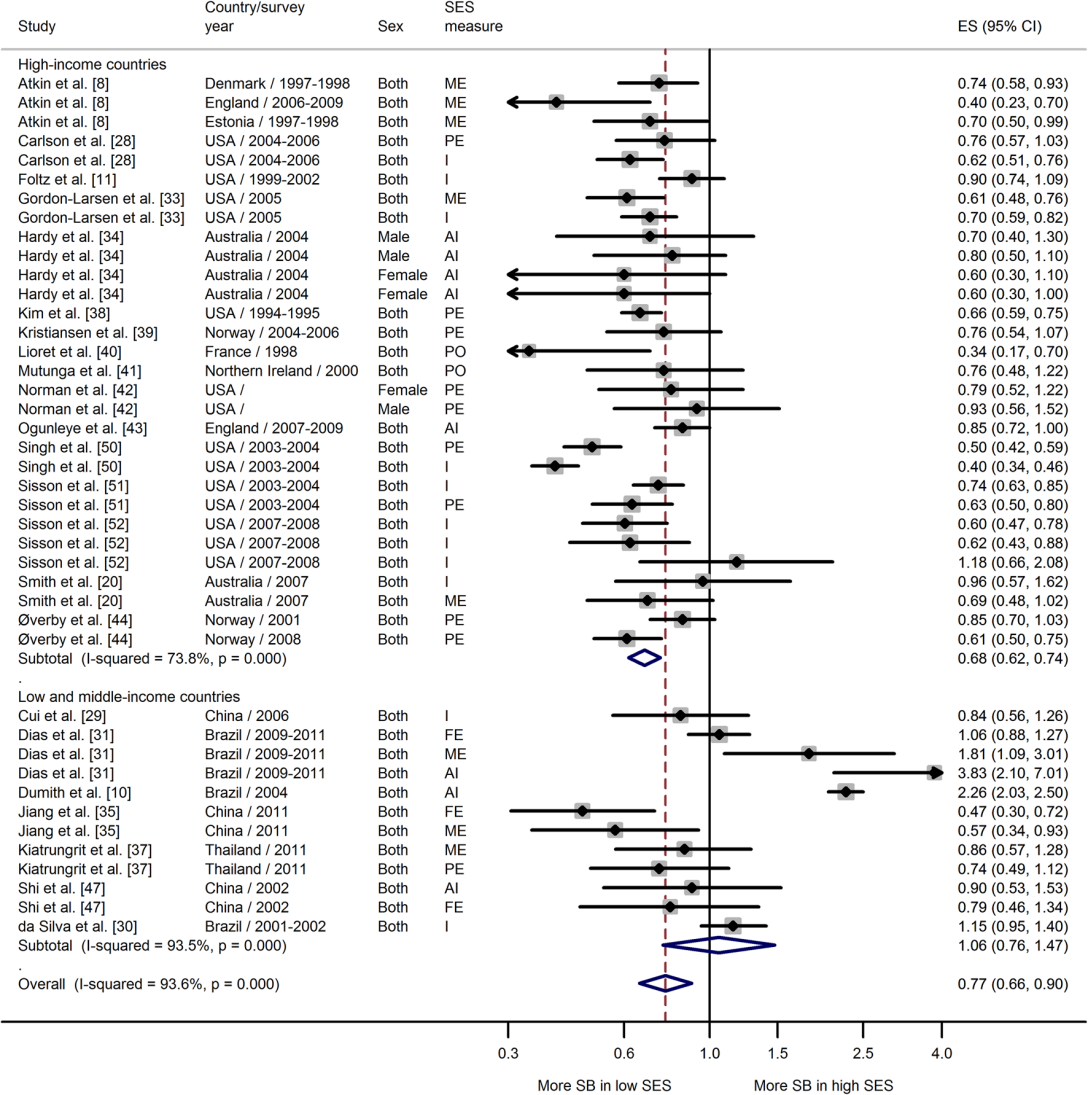
Meta-analysis of the association between socioeconomic status and high screen-based time. *AI* assets index, *CI* confidence interval, *ES* effect size, *FE* paternal education, *I* income, *ME* maternal education, *PE* parental education, *PO* parental occupation, *SB* sedentary behavior, *SES* socioeconomic status

**Fig. 4:**
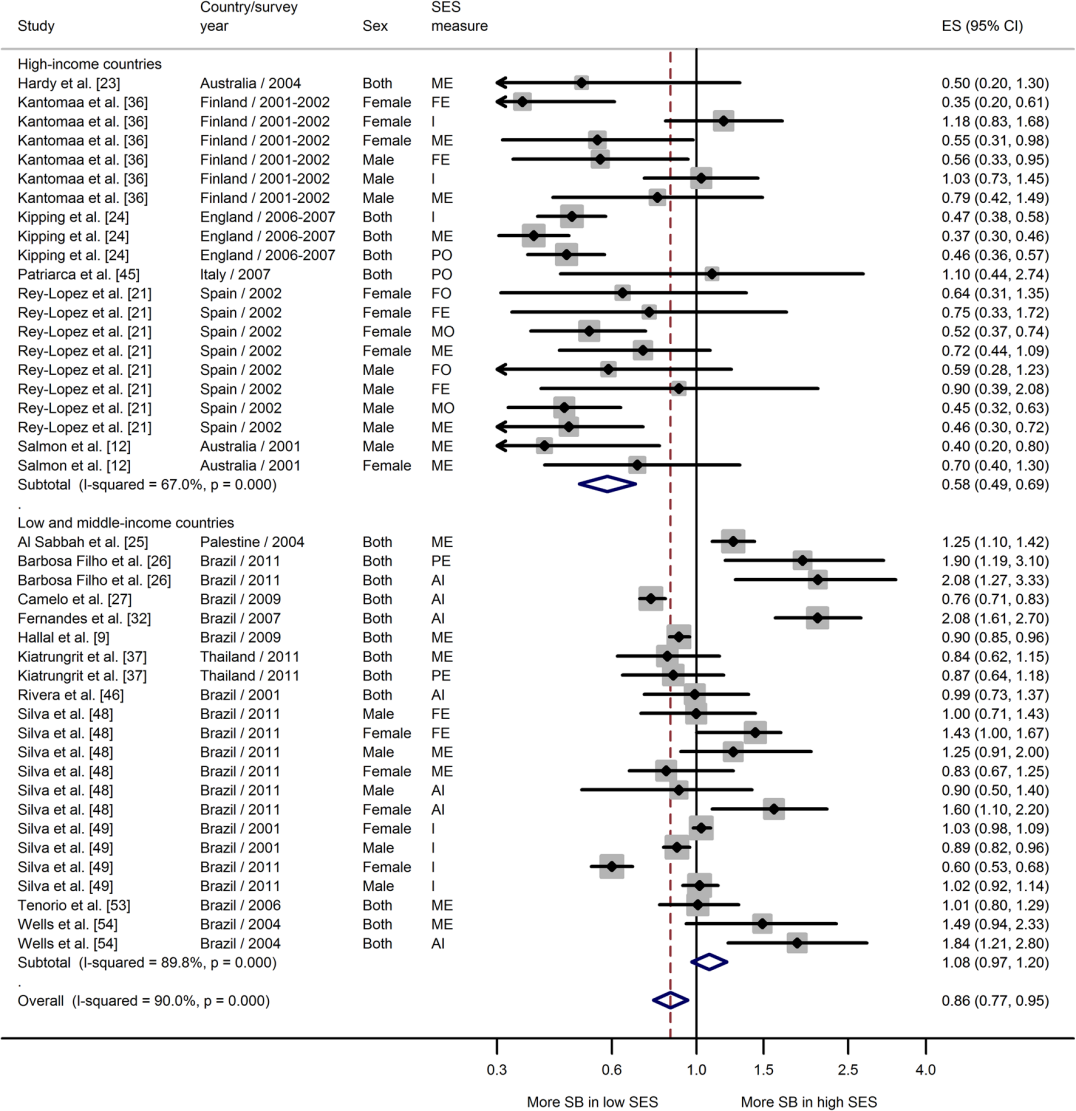
Meta-analysis of the association between SES and high television viewing time. *AI* assets index, *CI* confidence interval, *ES* effect size, *FE* paternal education, *FO* paternal occupation, *I* income, *ME* Maternal education, *MO* maternal occupation, *PE* parental education, *PO* parental occupation, *SB* sedentary behavior, *SES* socioeconomic status

**Fig. 5:**
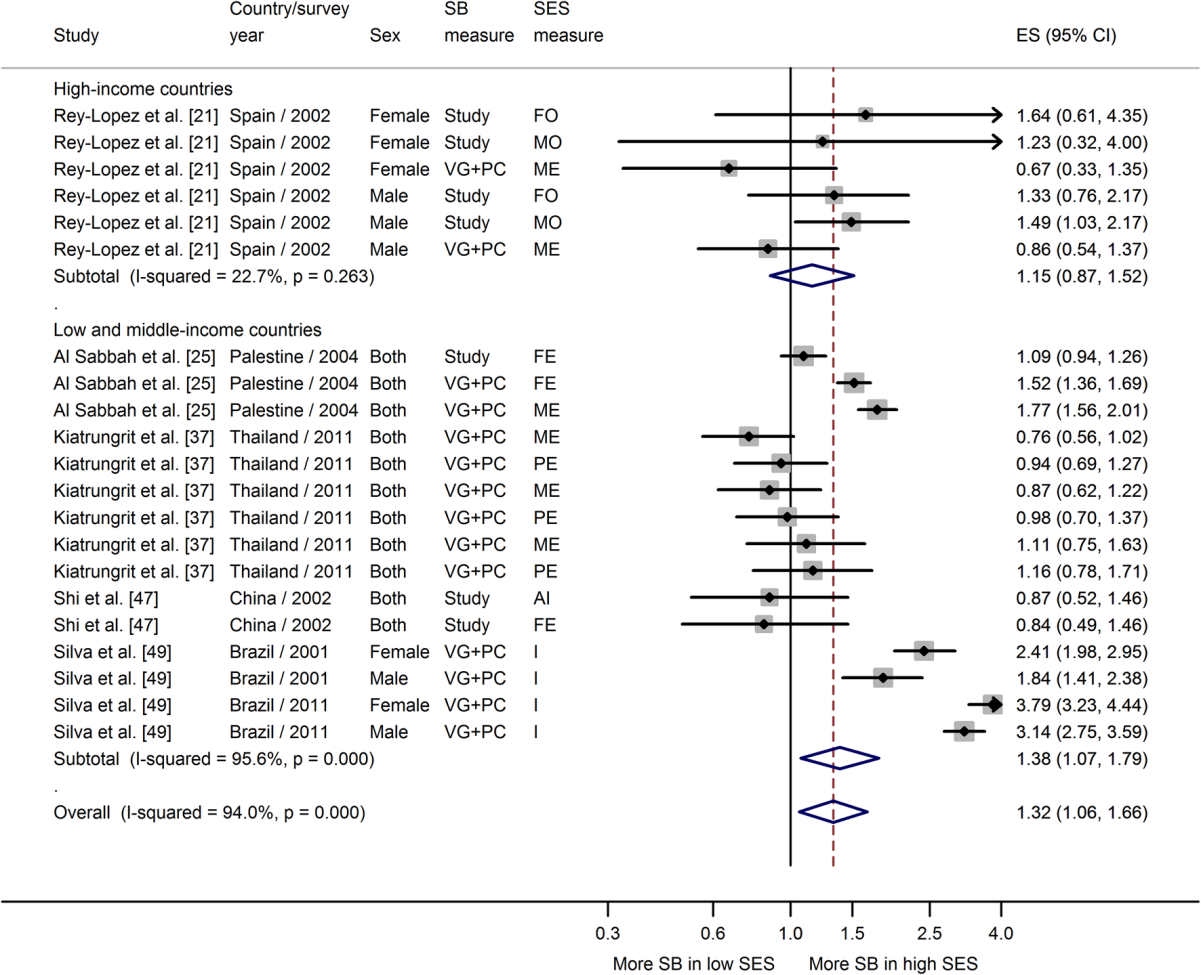
Meta-analysis of the association between socioeconomic status and other sedentary behavior domains (computer, video game, study time, but not including TV time). *AI* assets index, *CI* confidence interval, *ES* effect size, *FE* paternal education, *FO* paternal occupation, *I* income, *ME* maternal education, *MO* maternal occupation, *PC* computer, *PE* parental education, *SB* sedentary behavior, *SES* socioeconomic status, *VG* video game

Meta-analyses showing the associations between SES and each of three sedentary behavior measures, stratified by country income, are shown in Figs. 3, 4, and 5. Data from studies that combined TV, video, and computer game time showed a strong negative association between SES and high screen time in high-income countries (ES 0.68; 95 % CI 0.62–0.74) (Fig. 3). Of 30 individual estimates, half were significantly associated with lower SES and only one OR was greater than 1.00 (but not statistically significant). The same pattern was not observed among low-middle-income countries, where there was no association between SES and screen time (ES 1.06; 95 % CI 0.76–1.47). Heterogeneity among estimates of SES and screen time was higher in the studies from low-middle-income countries, with some differences in the direction of the association in studies from Brazil (positive) compared with those from China and Thailand (negative) (Fig. 3).

A similar pattern of association was found when only estimates of TV viewing time were examined (Fig. 4). There was a clear inverse association between SES and TV time in high-income countries (ES 0.58; 95 % CI 0.49–0.69), but no association between SES and TV time in low-middle-income countries (ES 1.08; 95 % CI 0.97–1.20). This latter finding reflected the greater heterogeneity in studies from low-middle-income countries.

The meta-analysis of data from studies that included a range of sedentary domains, but not including TV time (“other” sedentary time), is shown in Fig. 5. In contrast with the findings for screen and TV-viewing time, this analysis showed that adolescents with higher SES tended to spend more time in sedentary behavior than those with lower SES, irrespective of country income status. However, heterogeneity was high in the low-middle-income country studies, largely because of some very strong effects reported by one Brazilian study (Fig. 5).

A series of sensitivity analyses did not substantially change these results. Although the number of SES categories was not an important source of heterogeneity in the general meta-analysis (adjusted *R* ^2^ = −0.56 %), when the data were stratified by country income, there was a positive association between the number of SES categories included in the pooled-effect model and estimate of meta-analysis, but only in low-middle-income countries (OR_meta-regression_ 1.21; 95 % CI 1.02–1.44). The funnel plots and Egger’s tests showed no evidence of publication bias for the studies from either low-middle-income (*p* = 0.309) or high-income countries (*p* = 0.179). Influence analyses did not show important changes to the pooled-effect sizes due to any individual study in low-middle-income countries or in high-income countries.

## Discussion

Although several systematic reviews have already been conducted, this study was the first to quantify the associations between SES and sedentary behaviors in adolescents via meta-analysis techniques. By including data from more than 350,000 participants, we were able to calculate an overall pooled effect and examine the factors contributing to variations in the strength and direction of this association. Understanding the sources of variation in studies of adolescents is important, as it allows strategies for behavior change to be specifically targeted to this life stage. Overall, the pooled results from 39 studies showed that the odds of high sedentary behavior were 11 % lower in the highest SES groups than in the lowest SES groups. However, substantial heterogeneity existed, with contrasting findings in studies from high- and low-middle-income countries, for different domains of sedentary behavior, and—to a lesser extent—by the SES variable used.

There was a negative association between SES and sedentary behavior in adolescents from high-income countries (hence adolescents from low SES backgrounds were more likely to have high levels of sedentary behavior than their high-SES counterparts). The reverse was true in low-middle-income countries, where the higher SES adolescents were more likely to be highly sedentary. This contrast was underpinned by differences in domain-specific associations, with inverse associations between SES and screen and TV time in high-income countries and a positive association between SES and ‘other’ screen time in low-middle-income countries.

We found the associations between SES and sedentary behavior varied in the different country income groups, but this variation was underpinned by complex inter-relationships with both domains of sedentary behavior and measures of SES. Differences in access to TVs and computer games in high- and low-middle-income households may explain this finding, as studies in high-income countries have consistently found that adolescents from homes with more TVs and computers, and those with a TV in the bedroom, report more screen-based sedentary behavior [6]. However, access to TVs and video/computer games differs in low-middle-income countries. For example, between 2000 and 2012, the proportion of households in Brazil with a TV increased from 87 to 95 %, while the proportion with a computer increased from 10 to 39 %. These trends were strongly related to economic status, with better access to computers in wealthier households [19].

As adolescents in both country income groups seem to have wide access to TVs (but not necessarily to computers), another explanation for our main finding could be that, in low-middle-income countries, ownership of electronic devices and TVs is probably more determined by financial resources than education. For example, two Brazilian studies that included the effects of both education and resources have shown positive associations between income and sedentary behavior, but not between parental education and sedentary behavior [20, 21]. Indeed, our meta-analysis showed that the effect of resources on sedentary behavior in low-income countries was 38 % greater than the effect of parental education. In contrast, reviews have shown that both income and education are associated with screen-based sedentary behaviors, and that parental rules and limitations on screen time in families with higher levels of education were associated with less time spent in screen-based sedentary behavior [5, 6, 22].

One challenge in this study was the high level of variation in the sedentary behavior measures. We originally intended to develop a separate analysis for each sedentary behavior domain, but the small number of estimates for some domains, and the combinations of domains included in different measures of sedentary behavior, made this impossible. For example, only one study from a high-income country (Spain) [23] and two from low-middle-income countries (China [24] and Palestine [25]) provided estimates of study time (see ESM Fig. S1), and these were combined with video game and computer time (see ESM Fig. S2). The three broad categories of sedentary behavior used here—TV time, screen time (including TV, computer, and game time) and “other” screen time (i.e., not including TV)—showed different patterns in the SES association, which overall seemed to reflect the socioeconomic factors relating to access, either through availability of devices, or through parental control of behaviors [13, 15].

A second challenge for this meta-analysis was that the definitions of “high” sedentary behavior varied across studies. Although guidelines from the American Academy of Paediatrics suggest that adolescents should not spend more than 2 h per day in screen-based activities [26], several different cut-points were used in the original studies. However, more than half the estimates of high sedentary behavior were based on the 2 h/day limit, and different cut-points did not represent an important source of heterogeneity in the results. Our sensitivity analyses found the results were virtually the same when only those studies that used the 2 h/day cut-point were included. Furthermore, we chose not to include objective measures of sedentary behavior. Although we located studies of the association between SES and objectively measured sedentary behavior, cut-points used to define “sedentary” varied, and none of the studies provided a breakdown of time spent in different domains or a definition of “high” sedentary behavior, making it impossible to harmonize the data from objective and subjective measures. Another potential limitation was that we included separate estimates from studies that used more than one SES indicator. This may have introduced bias and a “narrowing” of the pooled estimates. However, our sensitivity analyses showed similar results when only one estimate from each study was included.

A third challenge was that the included studies defined the different SES variables with various numbers of categories, making it difficult to pool results and potentially leading to issues of misclassification. To minimize this, we included only the extreme groups reported in each study. Sensitivity analyses showed the results were unchanged when three categories were used. However, when four or more SES categories were included, we found stronger effect measures, mainly among low-middle-income countries.

As may be expected when attempts are made to combine the results of studies that used diverse methods of data collection and varying definitions of both SES and sedentary behaviors, there was marked heterogeneity in the findings. However, a strength of our study was that we attempted to explain this heterogeneity by conducting a series of meta-regression analyses with subgroups. High heterogeneity in the first analyses led us to investigate the variation in the association between SES and sedentary behavior in adolescents from countries with different income levels, and in different sedentary behavior domains, and using different measures of SES.

This study makes an important contribution to our understanding of associations between SES and sedentary behavior in adolescents, because most previous studies have only reported results based on the presence or absence of an association, with significance indicated by *p* values, without reporting the magnitude of the association. For example, one study whose results were included in this meta-analysis reported no “significant” associations (*p* < 0.05), but showed a strong OR, limited by a small sample size [27]. Another reported statistically “significant” findings based on very small differences in sedentary time (<5 % between the lowest and highest SES groups), but with very large samples (>60,000) [9].

The main limitation of this study is that interpretation of these findings, especially of the overall pooled estimates, is hampered to some degree by heterogeneity and other sources of potential bias. However, the absence of publication bias, the consistency of our results identified through sensitivity analyses, and use of a more conservative random-effects model for analysis enhance the confidence we have in our conclusions. We also conducted subgroup analyses to investigate whether results differed when education was measured using paternal, maternal, or parental education; no important differences were found. In England, Kipping et al. [28] investigated associations between SES, measured by social class, maternal education, and family income. They found that, after mutual adjustment for other SES variables, family income and maternal education were both inversely associated with TV viewing time [28].

A second limitation is that, with studies from only 15 different countries, the results cannot be extrapolated worldwide. Most of the studies from low-middle-income countries came from Brazil. However, significant differences exist in the cultural, social, and economic contexts of Brazil and China, which were grouped together for the purposes of this analysis because the World Bank classifies both as middle-income countries. These differences might affect the association between SES and sedentary behavior among adolescents. In addition, although our meta-analysis included peer-reviewed publications written in English, Spanish, and Portuguese, four studies in other languages (e.g., Arabic) had to be excluded because we could not translate them.

In terms of future research, data from prospective studies that focus on determinants rather than correlates will be useful. Objective measures of sedentary behaviors, with pattern recognition to identify domains, will also advance this field. However, a need remains for studies from low- and middle-income countries other than Brazil if future interventions are to address sedentary behaviors in socially and cultural relevant contexts.

## Conclusion

The findings of this review show that the relationships between SES and high sedentary behavior differ between high- and low-middle-income countries and vary by domain of sedentary behavior, and, to a lesser extent, by measure of individual SES. These complex associations between environmental, cultural, social, and individual factors and sedentary behaviors can inform the development of both local and population-based strategies that will support adolescents to choose activity over sedentariness whenever there is a possibility of choice. Our findings suggest that different approaches may be required when developing intervention strategies for reducing sedentary behavior in adolescents in different parts of the world.

## Electronic supplementary material

Below is the link to the electronic supplementary material.

Supplementary material 1 (DOCX 738 kb)
