# Supplementary material for: Socioeconomic Correlates of Sedentary Behavior in Adolescents: Systematic Review and Meta-Analysis
Source: Sports Med. 2016 Jun 3;47(1):61–75. doi: 10.1007/s40279-016-0555-4 (PMC5215067; doi:10.1007/s40279-016-0555-4)
Supplement: Supplementary file 1 — Supplementary material 1 (DOCX 738 kb) [file 40279_2016_555_MOESM1_ESM.docx]

**Electronic Supplementary Material**

Electronic Supplementary Material Table S1: Overall meta-analysis showing heterogeneity sources and odds ratios for the associations between SES and high sedentary behavior variables.

| **Variables** | n^a^ | ES pooled (95% CI) | I^2^ | Meta-regression  OR (95% CI) | %  heterogeneity  explained (R^2^) |
| --- | --- | --- | --- | --- | --- |
| **Sedentary behaviour definition** |  |  |  |  |  |
| Screen time ^b^ | 24 | 0.86 (0.69-1.07) | 94.3 | Index | 23.1 |
| Study | 7 | 1.12 (0.99-1.27) | 0.0 | 1.34 (0.88-2.02) |  |
| TV | 42 | 0.85 (0.77-0.94) | 90.0 | 0.99 (0.78-1.24) |  |
| TV+ ^c^ | 18 | 0.66 (0.57-0.76) | 81.6 | 0.77 (0.58-1.01) |  |
| VG+PC | 12 | 1.34 (0.93-1.92) | 95.6 | 1.64 (1.23-2.19) |  |
| **SES** |  |  |  |  |  |
| Paternal occupation | 4 | 0.94 (0.58-1.54) | 45.1 | Index | 5.2 |
| Paternal education | 11 | 0.83 (0.67-1.04) | 75.7 | 0.86 (0.45-1.62) |  |
| Income | 22 | 0.99 (0.79-1.24) | 97.9 | 1.05 (0.58-1.91) |  |
| Maternal occupation | 4 | 0.76 (0.40-1.46) | 88.3 | 0.79 (0.37-1.69) |  |
| Maternal education | 27 | 0.78 (0.68-0.89) | 83.2 | 0.82 (0.45-1.49) |  |
| Parental education | 15 | 0.78 (0.68-0.88) | 71.5 | 0.84 (0.46-1.55) |  |
| Parental occupation | 2 | 0.63 (0.28-1.45) | 70.0 | 0.65 (0.26-1.62) |  |
| SES index | 16 | 1.14 (0.85-1.53) | 95.7 | 1.22 (0.66-2.25) |  |
| Social class | 2 | 0.53 (0.24-1.17) | 71.1 | 1.57 (0.26-1.44) |  |
| **Country income ^d^** |  |  |  |  |  |
| Low-middle income | 47 | 1.15 (1.01-1.30) | 95.8 | Index | 35.0 |
| High-income | 56 | 0.67 (0.62-0.73) | 75.3 | 0.59 (0.50-0.69) |  |
| Total | 106 | 0.87 (0.80 to 0.96) | 94.5 | - | - |

a-represents the number of estimates available;

b- Estimates based on studies that measured sedentary behaviour as time spent in television + computer + video games + other screen-based activities;

c- Estimates based on studies that measured sedentary behaviour as time spent in computer, video game, study time, but not including TV time

d- according to World Bank classification;

SES: socioeconomic status; VG: video game; PC: computer; SES; Socioeconomic status; ES: effect size; 95%CI: 95% confidence interval.ES: effect size; OR: odds ratio; CI: Confidence interval;


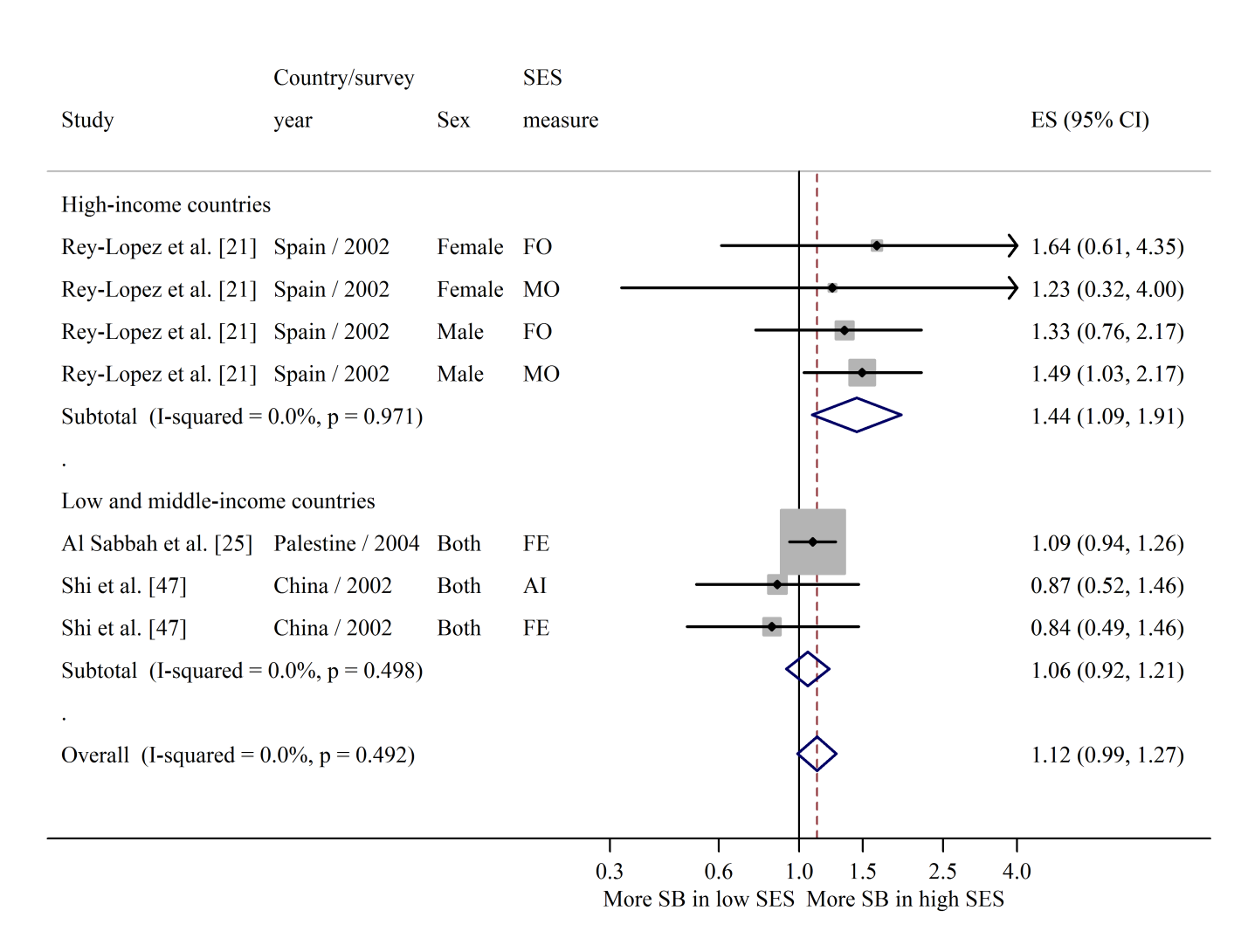


**Electronic Supplementary Material Figure S1: Meta-analysis of the association between SES and high studying time.** (FE: Paternal education; AI: Assets index; FO: Paternal occupation; MO: Maternal occupation). SB: Sedentary behaviour; SES; Socioeconomic status; ES: effect size; 95%CI: 95% confidence interval.

**
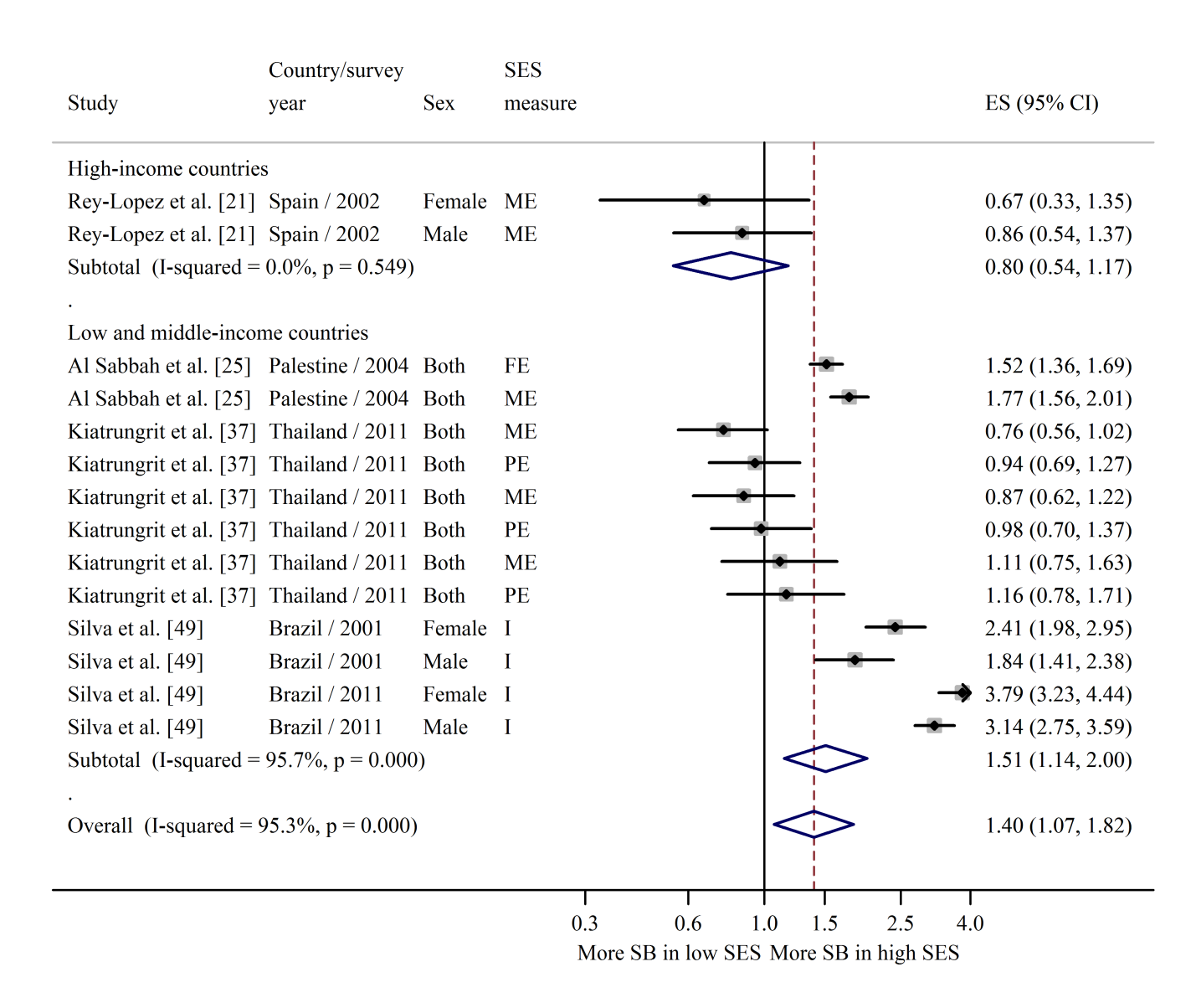
**

**Electronic Supplementary Material Figure S2: Meta-analysis of the association between SES and high computer and video game time.** (ME: Maternal education; PE: Parental education; I: Income). SB: Sedentary behaviour; SES; Socioeconomic status; ES: effect size; 95%CI: 95% confidence interval.
